# Clinical results of conformal versus intensity-modulated radiotherapy using a focal simultaneous boost for muscle-invasive bladder cancer in elderly or medically unfit patients

**DOI:** 10.1186/s13014-016-0618-6

**Published:** 2016-03-18

**Authors:** Lotte J. Lutkenhaus, Rob M. van Os, Arjan Bel, Maarten C. C. M. Hulshof

**Affiliations:** Department of Radiation Oncology, Academic Medical Center, Meibergdreef 9, 1105 AZ Amsterdam, The Netherlands

**Keywords:** Radical radiotherapy, Focal boost, Bladder cancer, Intensity-modulated radiotherapy, Toxicity

## Abstract

**Background:**

For elderly or medically unfit patients with muscle-invasive bladder cancer, cystectomy or chemotherapy are contraindicated. This leaves radical radiotherapy as the only treatment option. It was the aim of this study to retrospectively analyze the treatment outcome and associated toxicity of conformal versus intensity-modulated radiotherapy (IMRT) using a focal simultaneous tumor boost for muscle-invasive bladder cancer in patients not suitable for cystectomy.

**Methods:**

One hundred eighteen patients with T2-4 N0-1 M0 bladder cancer were analyzed retrospectively. Median age was 80 years. Treatment consisted of either a conformal box technique or IMRT and included a simultaneous boost to the tumor. To enable an accurate boost delivery, fiducial markers were placed around the tumor. Patients were treated with 40 Gy in 20 fractions to the elective treatment volumes, and a daily tumor boost up to 55–60 Gy.

**Results:**

Clinical complete response was seen in 87 % of patients. Three-year overall survival was 44 %, with a locoregional control rate of 73 % at 3 years. Toxicity was low, with late urinary and intestinal toxicity rates grade ≥ 2 of 14 and 5 %, respectively. The use of IMRT reduced late intestinal toxicity, whereas fiducial markers reduced acute urinary toxicity.

**Conclusions:**

Radical radiotherapy using a focal boost is feasible and effective for elderly or unfit patients, with a 3-year locoregional control of 73 %. Toxicity rates were low, and were reduced by the use of IMRT and fiducial markers.

**Electronic supplementary material:**

The online version of this article (doi:10.1186/s13014-016-0618-6) contains supplementary material, which is available to authorized users.

## Background

Standard therapy for muscle-invasive bladder cancer is radical cystectomy with bilateral lymph node dissection [1], providing 3-year recurrence-free survival rates of 60–68 % [2–5]. Bladder-preserving strategies, such as trimodality treatments combining radiochemotherapy with a transurethral resection of the bladder tumor (TUR-B) are usually only offered to patients who refuse cystectomy or who are considered inoperable [6–8]. The superiority of surgery over a bladder-preserving strategy has not been proven in a randomized trial, although long-term data shows that overall survival for both strategies is comparable [1, 9–11]. It has been shown that a combination of radiotherapy and chemotherapy results in a higher locoregional control and overall survival compared to radiotherapy alone [6, 12, 13], resulting in radiochemotherapy being the preferred bladder-sparing treatment option. However, patients who are referred for bladder-sparing approaches are mostly elderly or unfit, which regularly also contraindicates chemotherapy [14]. This leaves the combination of TUR-B and radical radiotherapy as the only treatment option.

Radiotherapy techniques have improved over the past years, from box techniques incorporating the entire pelvis, to adaptive strategies combined with rotational delivery reducing normal tissue doses [15–21]. For radical radiotherapy delivered with large, non-modulated treatment fields for both the bladder and the tumor boost area, without using daily image-guidance, relatively low three-year local control rates of 53–56 % have been reported [22–24]. For these radiotherapy treatments, with tumor doses between 55 and 70 Gy, acute and late toxicity rates grade ≥ 2 ranged between 20–67 %, and 7–17 %, respectively [22, 25–29]. More conformal treatment plans have shown to result in reduced toxicity [26], and seem promising in terms of local control when combined with daily image-guidance [30–32]. However, these studies consist of small patient numbers, and frequently have a short follow-up.

The aim of the present retrospective study was to analyze the treatment outcome and associated toxicity of conformal versus intensity-modulated radiotherapy, without concurrent chemotherapy, using a focal simultaneous boost for muscle-invasive bladder cancer in patients considered medically or technically inoperable.

## Methods

From 2003 to 2013, 132 patients with a muscle-invasive urothelial cell carcinoma of the bladder were treated with radical radiotherapy at the Academic Medical Center, The Netherlands. Of these, 8 patients were also included in a previous analysis [23]. Inclusion of patients was started upon implementation of an adaptive strategy [17], which yielded the range of used treatment techniques as homogeneous as we could achieve. Patients with multiple tumors were excluded, resulting in 118 patients available for analysis. The results were evaluated retrospectively. Patient and treatment characteristics are shown in Table 1. Median age at start of radiotherapy was 80 years (range: 41–95 years). All patients had a histologically diagnosed solitary T2-4 N0-1 M0 bladder tumor, and were inoperable, refused surgery or were medically unfit for radical surgery due to age or comorbidities. Staging procedures included clinical and digital rectal examination, chest X-ray, a pelvic and abdominal CT scan and cystoscopy. All patients underwent TUR-B prior to radiotherapy. Tumors were scored as T3 when a mass was palpated after the TUR-B, or when the CT scan revealed tumor extension in the perivesical fatty tissue. When muscle infiltration depth was unknown, the tumor was scored as T2.

**Table 1:** Patient and treatment characteristics

Characteristics	Patients	
	*n*	(%)
Sex		
Female	29	(25)
Male	89	(75)
WHO performance status		
0	13	(11)
1	67	(57)
2	35	(30)
3	3	(2)
Tumor stage^a^		
2	37	(31)
3	71	(60)
4	10	(9)
Histological grade		
2	11	(9)
3	107	(91)
Clinical lymph node involvement^b^		
No	109	(92)
Yes	9	(8)
Hydronephrosis		
No	97	(82)
Yes	21	(18)
Tumor size		
2–4 cm	37	(31)
4–6 cm	58	(49)
≥6 cm	22	(19)
Unknown	1	(1)
Tumor resection status		
Not resected	2	(2)
Complete resection	13	(11)
Incomplete resection	49	(41)
Unknown	54	(46)
Planned radiotherapy dose		
55 Gy	61	(52)
60 Gy	57	(48)
Radiotherapy technique		
3D-conformal	67	(57)
IMRT	43	(36)
VMAT	8	(7)
Focal simultaneous boost		
Concomitant	101	(86)
Simultaneously integrated	17	(14)
Treated with image-guidance		
No	42	(36)
Yes	76	(64)

^a^According to UICC (TNM) classification

^b^Patients with positive lymph nodes were not referred for radical radiotherapy. However, patients with one clinically dubious but not pathologically proven local node were included

### Radiotherapy

Patients were treated in 20 daily fractions, over a period of 4 weeks. The prescription dose to the elective area, i.e. bladder, prostate and pelvic lymph nodes, was 40 Gy. Lymph nodes were excluded from the elective field in case of comorbidities that required a target volume reduction. The tumor received a simultaneous boost of 0.75 Gy, delivered either concomitantly or simultaneously integrated with the elective dose. Before 2006, a total prescription dose of 55 Gy to the tumor was used. After 2006, a dose of 60 Gy was chosen, unless this would result in a too high small bowel dose (bowel volume receiving 60 Gy > 3 cm^3^). It was chosen to increase the tumor dose, since a previously conducted study at our institute showed a low toxicity profile for the 55 Gy schedule [23], leaving room for dose escalation and taking into account that no concurrent chemotherapy was administered. The additional 5 Gy dose was delivered in two fractions of 2.5 Gy at the end of treatment.

A CT scan with contrast filling for the bowel was acquired prior to treatment and used for planning purposes. Patients were instructed to have a full bladder during CT scanning and during treatment, by drinking 250 ml of fluid 1.5 h prior to treatment, and refrain from voiding in order to minimize the volume of non-involved bladder tissue receiving the boost dose. The bladder and gross tumor volume (GTV) were delineated on the planning CT scan. From 2004 onwards, GTV delineation was often aided by the cystoscopic placement of fiducial markers (used in 64 % of patients). At first, titanium clips were used [33], which were replaced by lipiodol in 2006 [34]. At the introduction of intensity-modulated radiotherapy (IMRT), the pelvic lymph nodes, prostate, rectum, small bowel cavity and femoral heads were also delineated on the planning CT scan. Three different planning techniques have been used between 2003 and 2013, which are described below. Table 1 lists the number of patients treated with each radiotherapy planning technique. Table 2 provides an overview of the used techniques.

**Table 2:** Treatment planning and delivery methods

	Elective			Boost^a^	
	Dose	Target organs	PTV	Dose^b^	Delivery
*3D-conformal*	40 Gy	Bladder, prostate, and pelvic lymph nodes^c^	Box technique, based on anatomical landmarks	55–60 Gy	Concomitant
*IMRT*	40 Gy	Bladder, prostate, and pelvic lymph nodes^c^	Cranially and anteriorly: 15 mm.	55–60 Gy	Concomitant
			Other directions: 8 mm	55–60 Gy	Simultaneously integrated^d^
*VMAT*	40 Gy	Bladder, and pelvic lymph nodes^c^	Cranially and anteriorly: 13 mm.	55–60 Gy	Simultaneously integrated
			Other directions: 7 mm		

^a^In case fiducial markers were present, a uniform boost margin of 10 mm was used. Otherwise, an adaptive margin strategy was employed

^b^A dose of 60 Gy was standard after 2006. 55 Gy was chosen only when a dose of 60 Gy would result in a too high small bowel dose

^c^Lymph nodes were excluded from the elective field in case of comorbidities that required a target volume reduction

^d^Simultaneous integration of the boost plan with the elective plan was implemented after October 2011

Before 2009, a conformal four-field box was used for the elective field, and the concomitant boost was delivered using 2–4 conformal beams. The cranial limit of the elective field was the L5-S1 interspace and the caudal limit was 5 mm caudal of the symphysis. Lateral margins were 10 mm beyond the maximal width of the bony pelvis, whereas the anteroposterior margins on the lateral fields were 15 mm beyond the bladder. The planning target volume (PTV) for the tumor, i.e. PTV_boost_, was obtained by expanding the GTV with a 15–20 mm margin in case no fiducial markers were present, as opposed to 10 mm in case of markers. When markers were not present, an adaptive margin strategy for PTV_boost_ was used, for which the GTV was redelineated on daily repeat CT scans acquired during the first week [17]. A summation of all GTV delineations was then expanded with 10 mm to create a second PTV_boost_, which was used from the second week onwards. Weekly offline position verification was performed using electronic portal images, which was replaced by cone beam CT (CBCT) in 2007.

From 2009 onwards, patients were treated with an IMRT technique. For this, a PTV_elective_ was created by expanding the combined bladder, prostate and lymph node delineations with 15 mm in cranial and anterior directions, and 8 mm in all other directions. PTV_boost_ was created as described before. Before October 2011, patients were treated with two separate IMRT-plans with 5 or 7 beams, for both the PTV_elective_ and the concomitant PTV_boost_ [15]. After October 2011, the boost dose was delivered simultaneously integrated with the elective dose. Standard planning objectives were used to obtain a target coverage of 95 % of the prescribed dose to 99 % of the PTV, while keeping dose to the organs at risk as low as possible. The introduction of IMRT was accompanied by the introduction of daily image-guidance using CBCT scans.

In 2012, rotational delivery of IMRT, i.e. volumetric modulated arc therapy (VMAT), was implemented at our department. Margins to create PTV_elective_ were reduced to 13 mm cranially and anteriorly, and 7 mm in all other directions, and the prostate was removed from the elective target volume for tumors not located in the bladder neck or prostatic urethra. The previously described adaptive margin strategy was used for PTV_boost_ in case no markers were present, otherwise a uniform margin of 9 mm was used. A daily simultaneously integrated dose of 0.75–1 Gy was delivered to PTV_boost_. Dual arc VMAT plans were created, and the same planning objectives were used as for IMRT.

### Follow-up

All patients that started treatment were included in the analysis of overall survival. Six patients who did not complete treatment were excluded from locoregional control analysis. Patients were seen by their radiation oncologist every week during the treatment course, 1 month after treatment, every 3 months thereafter for the first year, every 6 months thereafter up to 3 years, and once yearly up to 5 years. A cystoscopy was performed at 2 months after treatment and thereafter every 6 months. In case of locoregional symptoms or when endoscopically a non-complete response was observed, an additional cystoscopy or CT scan was performed. Complete response was defined as endoscopically no signs of vital tumor, whereas a partial response was defined as a tumor mass reduction > 50 %. All invasive and non-invasive recurrences in the bladder were scored as local progression. Urinary and intestinal toxicity were scored according to the Common Terminology Criteria for Adverse Events (CTCAE) version 4.0. Acute toxicity was scored as the maximum toxicity during treatment or the first 3 months thereafter, whereas late toxicity was the maximum toxicity occurring after 3 months. Bladder capacity was estimated by the patient, both before treatment and during follow-up. To this end, patients were asked to measure their maximum voiding volume at home, by voiding in a urinal or cup with volumetric indications. In addition, patients were asked during follow-up if their voiding capacity was improved or worsened compared to the initial capacity before treatment.

### Statistical analysis

All time intervals were calculated from the start of radiotherapy treatment. Locoregional control was defined as no histological proven nodal or bladder recurrence, whereas distant control was defined as no evidence of distant metastasis. Bladder-intact survival was defined as the survival without a muscle-invasive recurrence (either no recurrence or a successfully treated superficial recurrence) and without a salvage cystectomy. The Kaplan-Meier method was used to estimate survival. Possible predictors for survival and locoregional recurrence were examined in univariate cox proportional hazard regression analyses. Hazard ratios (HR) were calculated, with associated 95 % confidence intervals (CI) and p-values. For predictors with continuous values both a linear and non-linear (i.e. cubic spline) association with the specific outcome were tested. The toxicity scores were dichotomized into grade ≥ 1, grade ≥ 2 and grade ≥ 3. Possible predictors for toxicity were analyzed using logistic regression in case of predictors with continuous values, or the *χ*^2^ test for dichotomous predictors. The difference in pre- and posttreatment functional bladder capacity was tested using a paired *t*-test. A p-value < 0.05 was considered statistically significant, and statistical analysis was performed using R (version 3.1.0, The R Foundation for Statistical Computing, Vienna, Austria).

## Results

The median time of follow up was 23.7 months. First response measurements (*n* = 106) showed a complete response in 92 patients (87 %), and a partial response in 11 patients (10 %). One patient had stable disease, and 2 patients showed progression. Out of all 118 patients, 20 patients (17 %) developed a recurrence in the bladder during follow-up, of which 3 patients had a nodal recurrence. Four patients (3 %) developed a nodal recurrence without bladder recurrence, and 27 patients (23 %) developed a distal recurrence. Of the local recurring patients, 9 patients (45 %) experienced both a locoregional and distal recurrence. Six bladder recurrences were superficial and were treated with TUR-B and mitomycin C installations, whereas in the remaining 14 patients, local recurrences were muscle-invasive.

**Fig. 1:**
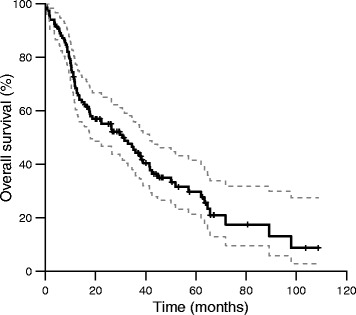
Overall survival (95 % confidence intervals depicted in grey dashed lines)

**Fig. 2:**
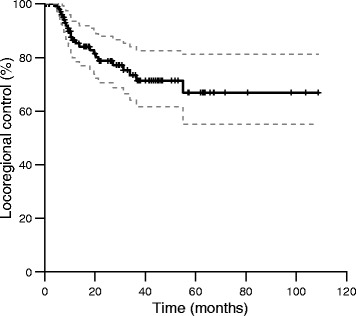
Locoregional control (95 % confidence intervals depicted in grey dashed lines)

The overall survival after 3 years was 44 % (95 % CI 36–55 %; Fig. 1). 37 patients died with bladder carcinoma, whereas 24 patients died from intercurrent disease. Two patients died from treatment complications. Bladder-intact survival after 3 years was 84 % (95 % CI 75–92 %). The 3-year locoregional and distant control rates were 73 % (95 % CI 64–84 %; Fig. 2) and 74 % (95 % CI 65–84 %), respectively.

On univariate analysis, a significant difference in overall survival or locoregional disease recurrence was not found for patients treated with either 3D-conformal radiotherapy or IMRT/VMAT. The only significant predictors for survival were age and not completing treatment (Tables 3 and 4). For locoregional disease recurrence, only the presence of hydronephrosis was a statistically significant predictive factor (Table 3). Locoregional control after 3 years was similar for patients receiving either 55 or 60 Gy, with 72 and 74 %, respectively (*p* = 0.55). The prognostic value for none of the predictors improved by assuming a non-linear relationship.

**Table 3:** Prognostic value for overall survival and locoregional recurrence of patient and tumor characteristics

Prognostic factors	Overall survival				Locoregional recurrence			
	*n*	HR	95 % CI	*p*	*n*	HR	95 % CI	*p*
Age	118	1.0	(1.0;1.1)	0.02	112	1.0	(0.95;1.0)	0.89
Tumor size	117	1.0	(0.90;1.2)	0.62	111	1.0	(0.76;1.3)	0.99
Residual mass after resection^a^								
Yes	57				52			
Possibly	27	0.81	(0.45;1.5)	0.49	27	0.83	(0.29;2.4)	0.73
No	16	0.47	(0.20;1.1)	0.09	15	0.80	(0.22;2.9)	0.73
Tumor location								
Not mobile part	27				25			
Mobile part	91	1.05	(0.61;1.8)	0.85	87	0.85	(0.34;2.1)	0.73
Clinical lymph node involvement								
No	109				103			
Yes	9	0.52	(0.19;1.4)	0.20	9	1.5	(0.44;5.0)	0.53
Hydronephrosis								
No	97				92			
Yes	21	1.29	(0.73;2.3)	0.38	20	2.9	(1.2;6.8)	0.01
Tumor stage								
T2-T3	108				103			
T4	10	1.05	(0.45;2.4)	0.91	9	0.52	(0.07;3.8)	0.23

NB. Due to exclusion of patients receiving less than their prescribed dose for the analysis on local recurrence, total amount of patients in this analysis is 112, which is different from the analysis on overall survival. In addition, residual mass after resection and tumor size were not known for all patients

^a^As assessed on the CT scan made for planning purposes

**Table 4:** Prognostic value for overall survival and locoregional recurrence of treatment characteristics

Prognostic factors	Overall survival				Locoregional recurrence			
	*n*	HR	95 % CI	*p*	*n*	HR	95 % CI	*p*
Received radiotherapy dose								
55 Gy	57				57			
60 Gy	55	0.70	(0.44;1.1)	0.15	55	0.81	(0.36;1.8)	0.61
< prescribed dose	6	5.3	(2.1;13.0)	<0.001				
Radiotherapy technique								
3D-conformal	67				63			
IMRT/VMAT	51	1.05	(0.66;1.7)	0.83	49	0.97	(0.43;2.2)	0.95
Treated with image-guidance								
No	42				40			
Yes	76	1.43	(0.88;2.3)	0.15	72	1.0	(0.45;2.4)	0.93
Elective lymph node irradiation								
No	24				23			
Yes	94	0.60	(0.36;1.0)	0.06	89	1.1	(0.37;3.1)	0.90
Use of fiducial markers								
No	43				40			
Yes	75	0.93	(0.59;1.46)	0.75	72	0.83	(0.37;1.9)	0.66

**Fig. 3:**
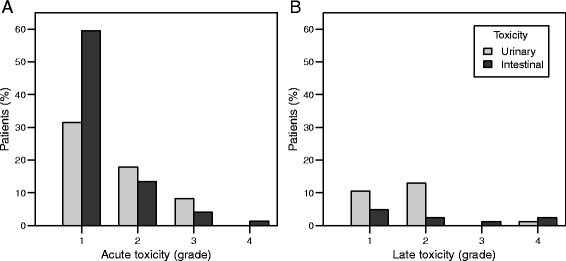
Acute and late toxicity. **a** Acute toxicity. **b** Late toxicity. Light bars represent urinary toxicity, dark bars represent intestinal toxicity

Acute intestinal and urinary toxicity was scored in 72 patients. Of these, 19 % experienced grade 2 or higher intestinal toxicity, compared to 26 % for urinary toxicity (see Fig. 3a). Late toxicity was scored in 100 patients. Late intestinal and urinary toxicity grade ≥ 2 was seen in 5 and 14 % of these patients, respectively (see Fig. 3b). Grade 3 intestinal toxicity was seen in 1 patient, and grade 4 in 2 patients. In 1 patient, grade 4 urinary toxicity was observed.

Univariate analysis revealed that the use of IMRT, combined with daily image-guidance, reduced late intestinal toxicity from 20 % for the conformal box technique, to 5 % for the intensity-modulated techniques (*p* = 0.05). In addition, the introduction of IMRT also influenced acute urinary toxicity (grade ≥ 3 toxicity reduced from 22 to 2 %, *p* = 0.02). Acute urinary toxicity was furthermore influenced by the use of fiducial markers, with a reduction in grade ≥ 2 toxicity from 53 % without markers, to 17 % with markers (*p* < 0.01). Logistic regression revealed a relationship between tumor size and acute intestinal and urinary toxicity, with tumor sizes of 2, 4, and 6 cm corresponding to risks of grade ≥ 1 acute intestinal toxicity of 45, 79, and 94 %, respectively (*p* < 0.01), and risks of grade ≥ 3 urinary toxicity of 1, 4 and 18 %, respectively (*p* = 0.04). Other characteristics were not predictive for either late intestinal or urinary toxicity (see Additional file 1: Table S1-S3).

Of the 96 patients for which information about bladder function is available, 92 % reported a stable or improved bladder function. Functional bladder capacity increased significantly, from a median of 200 ml before, to 250 ml after treatment (*p* = 0.004).

## Discussion

In the present study, we found a 3-year overall survival of 44 % for patients treated with TUR-B and radiotherapy with a focal simultaneous boost for bladder cancer, with a 3-year locoregional control of 73 %. Of the patients that were still alive after 3 years, 83 % had an intact bladder. We found acute urinary and intestinal toxicity rates grade ≥ 2 of 26 and 19 %, respectively, whereas late urinary and intestinal toxicity grade ≥ 2 was 14 % and 5 %, respectively. Toxicity rates were lower for patients treated with IMRT or fiducial markers around the tumor.

Our results are similar compared to a multicenter phase III trial by James et al., in which 360 patients were randomly assigned to undergo radiotherapy either with or without synchronous chemotherapy [6]. When comparing the radiotherapy-only results from their study with the current study, similar 3-year overall survival rates were found (Fig. 1) [6]. However, the results from chemoradiation as reported by James et al. compare favorably to our results, with a 3-year overall survival for chemoradiation of 57 %, compared to 44 % for the present study (Fig. 1). Our low survival rate probably reflects the selection of elderly or medically frail patients, who are likely to have a shorter survival independent of their bladder cancer or treatment. The 3-year locoregional control of 73 % found in the current study, compares favorably with other studies reporting on patients treated with radiotherapy only, which present rates of 53–64 % [22, 25, 27, 28]. The exclusion of patients that did not complete radiotherapy, and patients with multifocal tumors, will partly account for this effect. The higher accuracy of dose delivery, by means of fiducial markers and position verification with daily CBCT, may also contribute to the high locoregional control rate. Locoregional control was not influenced significantly by the tumor dose (55 versus 60 Gy), but tumor dose also did not influence toxicity. In the absence of concurrent chemotherapy, a tumor dose of 60 Gy is therefore still advocated, except when small bowel dose constraints are exceeded, in which case a dose of 55 Gy can be applied.

Other studies report late urinary and intestinal toxicity rates grade ≥ 2 of 10–17 %, and 7–10 % [22, 27–29], respectively. This is comparable to our results, with rates of 14 and 5 %, respectively. Since the risk of global bladder injury increases for doses over 50 Gy [35], the volume receiving the boost dose of 55 Gy should be as small as possible. This is reflected in our finding that the use of fiducial markers resulted in lower rates of acute urinary toxicity; when markers were used, the margins around the tumor were smaller, and a smaller volume of the non-involved bladder received 55 Gy. In addition, studies based on high-dose whole bladder irradiation as opposed to a tumor boost, report higher rates of late urinary toxicity grade ≥ 1: rates of 36–62 % have been reported, compared to 27 % we found [22, 28]. Regarding IMRT, we found a trend towards lower rates of grade ≥ 2 acute intestinal toxicity compared to a box technique (decrease from 33 % to 12 %, *p* = 0.06), which confirms the results of Søndergaard et al. [26]. This finding is in line with the lower bowel dose resulting from both the more conformal treatment technique, as well as the use of smaller margins [15, 16, 36]. The overall rates of toxicity in the present series were lower than the series by Søndergaard et al. which could be explained by a lower dose to the bladder and lymph nodes (40 versus 48–60 Gy). Since the volume of bowel receiving at least 45 Gy is most predictive for intestinal toxicity [26, 37], a dose prescription ≥ 40 Gy is expected to result in more intestinal toxicity.

The significant reduction found in both acute urinary and intestinal toxicity can be attributed to the introduction of fiducial markers, IMRT and daily image-guidance. Further increasing treatment accuracy, with the inherent reduction in margin size, could therefore further decrease toxicity. Adaptive strategies are vital in increasing treatment accuracy, since the interfractional movement of the bladder is a very large source of uncertainty. It has been shown that with an adaptive strategy, the dose to the bowel can be reduced while maintaining or improving target coverage [20, 21]. Further studies regarding outcome will determine whether this also results in a lower toxicity and a possibly higher local control.

Limitations of the present study are the retrospective nature of the data, and patient inclusion from a single institute. Our sample size was large compared to similar studies [22, 23, 25, 28], but was still relatively small concerning the univariate analyses on predictors for survival, recurrence and toxicity. For instance for survival, we only found age as predictor, whereas previously also tumor grade, tumor stage, performance status, complete resection and lymphatic invasion have been reported as predictors [10, 12, 29, 38]. In agreement with previous studies, we found hydronephrosis as a significant predictor for locoregional recurrence [9, 22, 28, 39]. It is possible that certain variables will prove to be predictors for several outcomes when a larger patient group is analyzed. Another limitation was the incomplete toxicity data, but this occurred randomly due to differences between physicians in the scoring of toxicity. A final limitation is the fact that patients treated with IMRT and VMAT were grouped together for univariate analyses. The PTV volume for VMAT is smaller since the introduction of VMAT coincided with the decision to exclude the prostate from the target volume, as well as a minor margin reduction, and this could have influenced the results. However, due to the small number of patients treated with VMAT, and the relatively small expected improvement in dose distribution [16], the effect on the analysis is assumed to be negligible.

## Conclusion

We found a 3-year overall survival of 44 % for patients treated with TUR-B and curative radiotherapy for bladder cancer. Three-year locoregional control was 73 %, with low rates of acute and late urinary and intestinal toxicity. Toxicity rates were reduced when using IMRT and fiducial markers. Radical radiotherapy using a focal simultaneous boost is therefore a feasible and effective treatment option for elderly or unfit patients with muscle-invasive bladder carcinoma, with a high change of preservation of bladder function.

## Additional file

Additional file 1: Table S1. Prognostic factors for toxicity grade ≥ 1. Table S2. Prognostic factors for toxicity grade ≥ 2. Table S3. Prognostic factors for toxicity grade ≥ 3. (PDF 251 kb)

## Abbreviations

CBCT: cone beam computed tomography
CI: confidence interval
CTCAE: common terminology criteria for adverse events
GTV: gross tumor volume
HR: hazard ratio
IMRT: intensity-modulated radiotherapy
PTV: planning target volume
TUR-B: transurethral resection of the bladder tumor
VMAT: volumetric modulated arc therapy
