# Supplementary material for: Clinical results of conformal versus intensity-modulated radiotherapy using a focal simultaneous boost for muscle-invasive bladder cancer in elderly or medically unfit patients
Source: Radiat Oncol. 2016 Mar 18;11:45. doi: 10.1186/s13014-016-0618-6 (PMC4797227; doi:10.1186/s13014-016-0618-6)
Supplement: Additional file 1: Table S1. — Prognostic factors for toxicity grade ≥ 1. Table S2. Prognostic factors for toxicity grade ≥ 2. Table S3. Prognostic factors for toxicity grade ≥ 3. (PDF 251 kb) [file 13014_2016_618_MOESM1_ESM.pdf]

Table S1: Prognostic factors for toxicity grade ≥ 1

Prognostic factors	Late toxicity					Acute toxicity				
		Urinary		Intestinal			Urinary		Intestinal	
	n	Tox. (%)	p	Tox. (%)	p	n	Tox. (%)	p	Tox. (%)	p
Tumor size	100		0.72		0.78	72		0.80		<0.01
Received radiotherapy dose										
55 Gy	44	32		16		29	48		79	1
60 Gy	51	24	0.50	10	0.53	38	66	0.23	77	
Elective lymph node irradiation										
No	17	35		19		21	52		86	
Yes	83	25	0.59	12	0.76	52	60	0.76	76	0.52
Use of fiducial markers										
No	34	38		12		19	68		90	
Yes	66	21	0.11	14	1	54	54	0.40	74	0.25
Radiotherapy technique										
3D-conformal	55	31		20		23	57		92	
IMRT/VMAT	45	22	0.46	5	0.05	50	58	1	72	0.11

Tox. = Toxicity.

Table S2: Prognostic factors for toxicity grade ≥ 2

**Prognostic factors**	**Late toxicity**					**Acute toxicity**				
		**Urinary**		**Intestinal**			**Urinary**		**Intestinal**	
	**n**	**Tox. (%)**	**p**	**Tox. (%)**	**p**	**n**	**Tox. (%)**	**p**	**Tox. (%)**	**p**
Tumor size	100		0.72		0.40	72		0.60		0.78
Received radiotherapy dose										
55 Gy	44	16		7		29	24		14	
60 Gy	51	12	0.77	4	0.84	38	26	1	23	0.52
Elective lymph node irradiation										
No	17	18		13		21	24		24	
Yes	83	13	0.93	4	0.40	52	27	1	17	0.73
Use of fiducial markers										
No	34	18		6		19	53		25	
Yes	66	12	0.65	5	1	54	17	0.006	17	0.63
Radiotherapy technique										
3D-conformal	55	15		7		23	30		33	
IMRT/VMAT	45	13	1	2	0.52	50	24	0.77	12	0.06

Tox. = Toxicity.

Table S3: Prognostic factors for toxicity grade ≥ 3

**Prognostic factors**		**Late toxicity**					**Acute toxicity**			
		**Urinary**		**Intestinal**			**Urinary**		**Intestinal**	
	**n**	**Tox. (%)**	**p**	**Tox. (%)**	**p**	**n**	**Tox. (%)**	**p**	**Tox. (%)**	**p**
Tumor size	100		0.62		0.38	72		0.04		0.34
Received radiotherapy dose										
55 Gy	44	2		2		29	14		3	
60 Gy	51	0	0.94	4	1	38	3	0.21	5	1
Elective lymph node irradiation										
No	17	6		6		21	14		14	
Yes	83	0	0.38	2	0.99	52	6	0.47	2	0.12
Use of fiducial markers										
No	34	0		6		19	26		10	
Yes	66	2	1	2	0.57	54	2	0.004	4	0.63
Radiotherapy technique										
3D-conformal	55	0		5		23	22		8	
IMRT/VMAT	45	2	0.92	0	0.34	50	2	0.02	4	0.82

Tox. = Toxicity.
